# Effect of Root Surface Biomodification on Multiple Recession Coverage with Modified Coronally Advanced Tunnel Technique and Subepithelial Connective Tissue Graft: A Retrospective Analysis

**DOI:** 10.3390/gels8010031

**Published:** 2022-01-04

**Authors:** Bartłomiej Górski, Marcin Szerszeń

**Affiliations:** 1 Department of Periodontal and Oral Mucosa Diseases, Medical University of Warsaw, 02097 Warsaw, Poland; gorskibartlomiej04@gmail.com; 2 Department of Prosthodontics, Medical University of Warsaw, 02097 Warsaw, Poland

**Keywords:** enamel matrix derivative (EMD), propylene glycol alginate (PGA), ethylenediaminetetraacetic acid (EDTA), modified coronally advanced tunnel technique, multiple gingival recessions, regeneration, subepithelial connective tissue graft

## Abstract

To improve treatment efficacy of gingival recessions (GR), chemical preparation of the exposed root surface was advocated. The aim of this study was to compare the additional influence of root biomodifications with 24% ethylenediaminetetraacetic acid (EDTA) alone or with enamel matrix derivative (EMD) on the 12 month outcomes of modified coronally advanced tunnel (MCAT) with subepithelial connective tissue graft in the treatment of multiple GR. Average root coverage (ARC), complete root coverage (CRC), reduction in GR, reduction in recession width (RW), gain in clinical attachment level (CAL), increase in gingival thickness (GT), increase in keratinized tissue width (KTW) and changes in root coverage esthetic score (RES) were evaluated. A total of 60 patients with 215 GR were enrolled. In 70, GR root surfaces were treated with EDTA + EMD, in other 72, with EDTA, while in the remaining 73 saline solution was applied. ARC was 94%, 89%, and 91% in the EDTA + EMD, the EDTA and the saline groups, respectively (*p* = 0.8871). Gains in clinical attachment level (CAL; 2.1 ± 1.1 mm) and RES values (9.6 ± 0.9) were significantly higher in the EDTA + EMD group, when compared with two other groups. The differences between other preoperative and postoperative parameters showed statistical significance only within but not between groups. MCAT outcomes may benefit from adjunctive use of EDTA + EMD regarding 12 month CAL gain and professionally assessed esthetics using RES following treatment of GR.

## 1. Introduction

Periodontitis is a chronic destructive inflammatory disease that affects soft and hard tissues surrounding and supporting the teeth; it has been associated with other systemic conditions such as type II diabetes, cardiovascular diseases, osteoporosis, premature birth, low birth weight, and rheumatoid arthritis [1]. A common soft tissue defect in daily clinical practice is gingival recession (GR), defined as root surface exposure due to migration of gingival margin apical to cemento-enamel junction (CEJ). This condition is widely associated with esthetic impairment, dentin hypersensitivity, and carious and non-carious cervical lesions [2]. 

Several surgical modalities have been developed for management of GR. Techniques vary in terms of different flap designs, with coronally advanced flap (CAF) and tunnel technique being used most widely for treatment of multiple GR [3]. The tunnel technique, first described by Zabalegui et al. [4], has been modified in recent years by several researchers with the use of full thickness flap preparation combined with application of subepithelial connective tissue graft (SCTG) and microsurgical approach [5,6,7]. The positive clinical and esthetic outcomes of modified coronally advanced tunnel technique (MCAT) may be attributed to flap elevation that preserves the integrity of papillae and avoids the use of vertical releasing incisions. Due to its conservative characteristics, MCAT potentially provides a better blood supply to the graft and wound stability, thus promoting faster healing [5,8]. 

In an attempt to improve treatment efficacy of GR, chemical preparation of the exposed root surfaces with numerous agents has been advocated. Application of 24% ethylenediaminetetraacetic acid (EDTA) gel (PrefGel^®^) (pH = 7) eliminates the smear layer, exposes collagen fibrils and unblocks dentinal tubules, all of which might enhance the attachment of the connective tissue [9]. In contrast, agents with a low pH, such as citric acid (pH = 1), can dissolve the collagen fibrous surface, leading to the formation of a granular dentin surface, which might have a deleterious effect on future attachment [10]. However, there is currently no consensus in the relevant academic literature whether root surface biomodification with EDTA improves clinical outcomes of soft tissue root coverage [11,12,13]. Enamel matrix derivative (EMD), on the other hand, accelerates migration, attachment, proliferation and differentiation of endothelial cells, periodontal ligament cells, cementoblasts and osteoblasts [14]. It is commercially available in a gel formulation containing porcine-derived enamel matrix proteins, propylene glycol alginate (PGA) and water (Emdogain^®^). Histologic observations in humans and animals have provided evidence for true periodontal regeneration with formation of new alveolar bone, acellular cementum and new attachment formation when EMD was applied [15,16,17]. Different studies have found beneficial effects of EMD application prior to root coverage procedures either with CAF or SCTG [18,19,20]. However, the role of EMD in periodontal plastic treatment with MCAT cannot be consistently evaluated and the extent to which outcomes of MCAT might be improved with EMD usage remains uncertain [5,7,21,22,23]. Moreover, before EMD application, the roots need to be conditioned for 2 min with a 24% EDTA to remove the smear layer, which may affect clinical outcomes.

Therefore, the aim of this study was to compare the potential additional influence of root surface biomodifications with EDTA alone or with EDTA + EMD, as compared to saline, on 12 month clinical and esthetic outcomes of MCAT with SCTG in the treatment of multiple gingival recessions of type 1 (RT1) and type 2 (RT2). The primary outcome variable was average root coverage (ARC) and complete root coverage (CRC). The secondary outcome variables were reduction in GR, reduction in recession width (RW), gain in clinical attachment level (CAL), increase in gingival thickness (GT), increase in keratinized tissue width (KTW) and changes in root coverage esthetic score (RES).

## 2. Results and Discussion

### 2.1. Results

A total of 215 gingival recessions were treated (70 defects in the EMD group, 72 defects in the EDTA group and 73 defects in the saline group), with similar inter-group characteristics and teeth distribution (Table 1).

At baseline, there were no significant differences in the clinical parameters between analyzed groups (Table 2). No patient presented adverse events or complications in healing during the follow-ups. All subjects kept scheduled appointments at their 12 month follow-up.

All three treatment modalities promoted a similar reduction in GR and RW and gain in CAL, KTW and GT. Statistically significant changes were observed in all groups at 12 months compared to baseline in all evaluated parameters. The corresponding values in terms of ARC measured at 12 months were 94 ± 00%, 89 ± 31% and 91 ± 23% in the EMD group, in the EDTA group and in the saline group, respectively. Post-treatment examination revealed that CRC for the EMD group at 12 months was achieved in 91%, in 90% in the EDTA group and in 89% of the saline group. The only significant difference observed between treatment modalities was related to CAL gain, which was higher in the EMD-treated sites (2.1 ± 1.0 mm) when compared with EDTA-treated sites (1.4 ± 1.1) and saline-treated sites (1.3 ± 1.0) at 12 months (*p* = 0.0216). The clinical results at baseline and 12 month visit are depicted in Table 2. 

The esthetic results after 12 months are presented in Table 3. The average RES in the EMD group was 9.65 ± 0.97, whereas in the EDTA group it was 8.88 ± 1.2 and 8.81 ± 1.30 in the saline group (*p* = 0.0091). Esthetic outcomes were significantly higher in the EMD group in terms of RES as well as three component parameters (soft tissue texture, marginal tissue contour and gingival color) when compared to the other two treatment modalities. Keloids did not form in any patient at any timepoint. 

### 2.2. Discussion

Factors potentially associated with clinical outcome after surgical treatment of GR can be divided into three categories: patient-dependent (plaque control, smoking, general health, compliance), preoperative site-specific characteristics (recession depth and width, presence of keratinized tissue, gingival thickness, and type of phenotype, loss of interproximal attachment, tooth type and tooth location, presence of frenula), and surgical procedures (flap design, root surface biomodifcation, type of graft, hyaluronic acid application) [24]. The purpose of this study was to evaluate whether the additional use of EDTA or EDTA + EMD in combination with MCAT + SCTG could provide superior outcomes of multiple RT1 and RT2 treatment at 12 months. Rinsing with saline solution was used as control, since studies have shown that saline is not able to remove the root smear layer [25]. The primary objective of this research was to assess percentage of root coverage at 12 months. To the best of our knowledge, this is the first study of this kind. Differences between preoperative and postoperative values in terms of GR reduction, ARC, CRC, KTW gain and GT gain were statistically significant only within but not between groups. ARC was 94%, 89%, and 91% in the EDTA + EMD, the EDTA, and the saline groups, respectively (*p* = 0.8871). CRC was achieved in 91%, 90%, and in 89% of cases in the EDTA + EMD, the EDTA and the saline groups, respectively (*p* = 0.9743). Differences between preoperative and postoperative values in terms of ARC, CRC, GR reduction, KTW gain and GT gain were statistically significant only within but not between groups. With respect to the aforementioned parameters, the present study confirms that root biomodification with either EDTA alone or with EDTA + EMD prior to root coverage procedures with MCAT + SCTG bears no significant influence on clinical outcomes. These observations prove that MCAT + SCTG is a highly effective method of multiple GR treatment, which is consistent with other studies [5,7,21,23] and a recent meta-analysis [26].

A notable finding of the present study was the significantly higher 12 month CAL gain in the EDTA + EMD group (2.13 ± 1.12 mm) when compared with the EDTA group (1.45 ± 1.10 mm) and the saline group (1.32 ± 1.03 mm). This outcome shows the influence of EMD on recession coverage and confirms the results of several previous studies. Recent clinical research comparing SCTG + EMD with SCTG alone in CAF showed that both methods were highly effective in root coverage with stable results over 24 months. However, after 36 months there were significantly better root coverage outcomes and higher amounts of keratinized tissue in the EMD group [27]. Another clinical study indicated that an EMD group showed less recession rebound and better recession coverage 2 years after CAF [28]. These findings suggest that CAL gain deteriorates over time after surgical intervention and there may be a beneficial effect of EMD on CAL stability. A recently published meta-analysis concluded that EMD provides higher CAL gain with moderate certainty evidence at 6 and 12 months following either CAF or CAF + SCTG [20]. However, there are little data in the literature on specific combination of EMD with MCAT. A clinical benefit of the application of EDTA + EMD to MCAT has been found in some trials [22], while others did not confirm such observations [5,23]. In our previous report, the likelihood of ARC > 85% increased sevenfold and of achieving CRC 21-fold, in favor of EDTA + EMD-treated sites [22]. Moreover, the 12 month CAL gain was significantly higher in EDTA + EMD sites (2.1 ± 1.0 mm) when compared with controls (1.6 ± 1.4 mm). Nevertheless, a study by Stähli et al. [23] found no benefits in terms of clinical parameters at a 6 month follow-up. Another study by Aroca et al. [5] found that the addition of EMD did not enhance clinical results after 12 months, but it included only teeth with Miller class III GR which overlaps RT2, while our study covered both RT1 and RT2 with a large majority of RT1 (67.1% EDTA + EMD; 68.0% EDTA; 69.8% saline). Having said that, it should be understood that blood contamination of the root surfaces might easily occur when MCAT is carried out, which may alter the effectiveness of EDTA root conditioning and the ability of EMD to precipitate and diminish its effectiveness [29]. By the same token, very recent studies reveal that adjunct application of EDTA alone might provide benefits in terms of GR reduction, and CAL gain when performing root coverage treatment with CAF + SCTG [11], but not in case of MCAT + SCTG [12]. 

Histological studies have revealed that the attachment between SCTG and the root surface was largely mediated by a combination of long junctional epithelium and connective tissue attachment, with connective tissue fibers running parallel to the root and little potential for new cementum and new bone formation [30]. The idea behind the application of root modifiers was to favor attachment of the regenerated periodontal structures to the root surface. It was assumed that with the smear layer removal and collagen fiber exposure, EDTA might stabilize the connection between the fibrin of the blood clot and the root surface [31,32]. In an in vitro study by Kasaj et al. [33], EDTA alone or in combination with EMD enhanced proliferation and density of fibroblasts. Furthermore, EMD enhanced extracellular matrix protein production and the level of transforming growth factor β that facilitated tissue repair and regeneration [9,34,35]. Shirakata et al. found that GR coverage with CAF + SCTG + EMD resulted in more periodontal regeneration and shorter junctional epithelium formation when compared with CAF + SCTG in dogs [36]. Human histological studies confirmed formation of new bone, cementum, and periodontal ligament on the tooth surface after using EMD in GR coverage [16,17]. Mercado et al. [27] hypothesized that the clinical benefit of having a periodontal attachment apparatus compared to a long junctional epithelium may be evident 3 years after surgical treatment. In light of this information, the biological basis for more CAL gain with the use of EMD in the current study might be explained by its biological properties in enhancing the attachment of connective tissue fibers to the root surface. 

The esthetic concern constitutes one of the main reasons for patients to seek recession treatment and thus highly esthetic outcomes are crucial for overall patient satisfaction [37]. At 12 months, total RES values were significantly higher for the EDTA + EMD group (9.66 ± 0.97) when compared with the EDTA and the saline group (8.88 ± 1.22 and 8.81 ± 1.30, respectively). As no differences were reported for CRC, which contributes to 60% of the RES score, differences in other soft tissue parameters were observed. Marginal tissue contour, soft tissue texture and gingival color were superior in the EDTA + EMD group. No significant differences were seen for two other treatment modalities. In this regard, enhanced esthetic outcomes might be attributed to adjunctive use of EMD, although the rationale for this finding is not entirely clear. However, EMD accelerates soft tissue wound healing and maturation, which could contribute to decreased scar tissue formation and improvements in soft tissue texture, marginal tissue contour, MGJ alignment and gingival color [38]. We previously described similar soft tissue response to root surface modification with either EDTA or EDTA + EMD [12,22], but no other researchers provided results that could be directly compared with ours. However, Aydinyurt et al. [39] assessed the impact of EMD application on esthetic outcomes of CAF + SCTG in treatment of Miller class I and II gingival recessions of contralateral maxillary canines. Despite the fact that no differences were observed between two treatment modalities in terms of total RES and CRC, the EMD group had significantly better results with regard to soft tissue texture and muco-gingival junction alignment. Although this clinical trial suggested some influence of EMD on esthetic outcomes, these results were obtained in different clinical cases (single versus multiple GR) with different surgical approaches (CAF versus MCAT).

The authors are aware of the limitations of the current study. There are some flaws associated with its retrospective nature, for example higher risk of bias. Nonetheless, all data of interest were available for the analysis, as they were collected from all patients. Moreover, as three treatment modalities were tested, a split-mouth model could not be applied. Another plausible limitation is the lack of histological evaluation of the healing pattern between the root surface and SCTG, but this was not possible for ethical reasons. Therefore, the assumption of better connective tissue attachment to the root surface with EMD was not verified. It is important to underscore that this methodological problem may limit our interpretations and prevent a definitive conclusion. Another potential drawback of this research stems from the fact that patient-reported outcome measures were not collected. Finally, as the distinct majority of GR defects were RT1, no separate statistical analyses were conducted for both recession types. However, a recent study supported the thesis that the distance from the tip of the papilla and the contact point and tooth location (maxilla versus mandible) are more important predictors for recession coverage than CAL [21]. A long-term follow-up of the presented patient population is intended as more differences between treatment modalities may arise with the increase of observation time. Further randomized clinical trials are required for assessing our results in terms of recurrence of GR. 

## 3. Conclusions

Within the limits of this 12 month study, it can be concluded that:

1. MCAT with SCTG was very effective in treating multiple gingival recessions regarding clinical outcomes independent of root surface modification,
2. EDTA + EMD may be beneficial in improving CAL gain at 12 months after root coverage with MCAT + SCTG,
3. The use of EDTA + EMD as an adjunct to MCAT + SCTG enhanced professionally assessed esthetic outcomes based on RES,
4. The use of 24% EDTA alone did not affect 12 month clinical and esthetic outcomes after MCAT + SCTG,
5. The present study confirms that root modification with either EDTA alone or with EDTA + EMD prior to root coverage procedures with MCAT + SCTG bears no significant influence on 12 month clinical outcomes (apart from CAL gain).

## 4. Materials and Methods 

### 4.1. Study Design 

This retrospective analysis of a randomized clinical trial included 60 consecutively treated subjects: 34 females and 26 males, whose mean age was 28.68 (range 21–37). The study was compliant with the Helsinki Declaration of 1975 as revised in Tokyo in 2004, reviewed and approved by the Bioethics Committee of Medical University of Warsaw (KB/208/2017) and registered with ClinicalTrials.gov (accessed on 27 November 2017) (NCT03354104). The subject population was recruited among patients referred to the Department of Periodontology and Oral Mucosa Diseases of Medical University of Warsaw between January 2018 and April 2020. Patients with multiple GR were qualified into the study by one examiner (TK) and each patient signed an informed consent form. They were instructed to use the roll tooth brushing technique and provided with dental prophylaxis and polishing. In each patient, GR in one quadrant was treated using SCTG with MCAT. In 20 subjects, root surfaces were modified with 24% EDTA and EMD, in other 20, with 24% EDTA alone, while in the remaining, saline solution was applied. Patients were followed for 12 months. 

### 4.2. Patient Population 

The inclusion criteria for the selected patients were: (1) at least three adjacent gingival recessions of type I (RT1) and/or II (RT2) at least 1 mm deep in maxillary or mandibular teeth [40]; (2) full-mouth plaque score (FMPS) <20% [41]; (3) full-mouth bleeding on probing (FMBOP) <20% [42]; (4) detectable cementoenamel junction (CEJ); and (5) minimum age of 18. The exclusion criteria were: (1) active periodontal disease; (2) caries lesions or restorations in the cervical area; (3) systemic diseases affecting healing potential or infectious diseases; (4) use of medications affecting periodontal status; (5) smoking; and (6) pregnancy or lactation.

Using improvements in percentage of root coverage as the primary outcome variable and assuming that the standard deviation of differences in measurements would not exceed 30% [43], the sample size for comparing differences in the three groups was calculated to be 12 subjects per treatment group. This would provide 80% power to disclose a true difference of 20 percentage points between tests and controls. However, 20 patients were included in the study to account for possible dropouts. 

### 4.3. Clinical Examination 

Clinical parameters were recorded at baseline and 12 months after surgery by one single blinded examiner (MS). A total of six patients not included in the study with at least three GR were used to calibrate the examiner, who assessed all GR in each patient with an interval of 24 h between recordings. Calibration was accepted when ≥90% of the recordings were reproduced within a difference of 1.0 mm, and exact measurements were repeated in 75% of the recordings. A graded periodontal probe (UNC probe 15 mm, Hu-Friedy, Frankfurt, Germany) was used for clinical measurements under local anesthesia. The values were rounded off to the nearest 0.5 mm. The parameters evaluated in the study were: (1) gingival recession height (GR) from free gingival margin to CEJ; (2) RW at the level of CEJ; (3) probing pocket depth (PPD) from free gingival margin to the bottom of gingival sulcus; (4) CAL from CEJ to the bottom of gingival sulcus; (5) KTW from the free gingival margin to the muco-gingival junction (MGJ); (6) GT measured 3 mm apical to the gingival margin with the use of endodontic spreader 25 ISO (Poldent, Warsaw, Poland) and a silicon stopper positioned perpendicularly to the gingival surface until alveolar bone or root surface was reached. 

### 4.4. Esthetic Evaluation 

A blinded examiner (MS) evaluated the esthetic outcomes according to RES at 12 months [44]. Briefly, a score of 0, 3, or 6 was used for the evaluation of gingival margin position, whereas a score of 0–1 was used for each of the other variables (marginal tissue contour, soft tissue texture, mucogingival junction alignment, and gingival color). The highest esthetic score to be achieved was 10. 

### 4.5. Surgical Phase 

All surgical procedures were performed by the same periodontist (BG) in accordance with the MCAT technique as described by Zuhr et al. [6]. GR were randomly assigned to treatment modality by means of a random number generator software prior to surgery; this was done by a statistician not involved in the study. Allocation of treatment was concealed in sealed and opaque envelopes and was revealed to the operator immediately before the surgery. No information on treatment allocation was provided to the patient. 

After local anesthesia with 4% articaine hydrochloride with adrenaline (1:100,000) (Ubistesin Forte 1.7 mL, 3-M ESPE, Saint Paul, MN, USA), the exposed root surfaces were mechanically treated with Gracey curettes (Hu-Friedy, Chicago, IL, USA). Full-thickness flap was raised up to MGJ and subsequently a split-thickness flap was prepared beyond MGJ with supraperiosteal dissection. The papillae were detached in their buccal aspects with the periosteum. Subsequently, SCTG was harvested from the palate as the free gingival graft and was subsequently de-epithelialized achieving the thickness of less than 1 mm and width around 4 mm. The donor site was covered with hemostatic sponge stabilized with cross-mattress non-resorbable sutures (Seralon 4/0 18 mm 3/8, Serag-Wiessner GmbH & Co. KG, Neila, Germany). 

**Figure 1:**
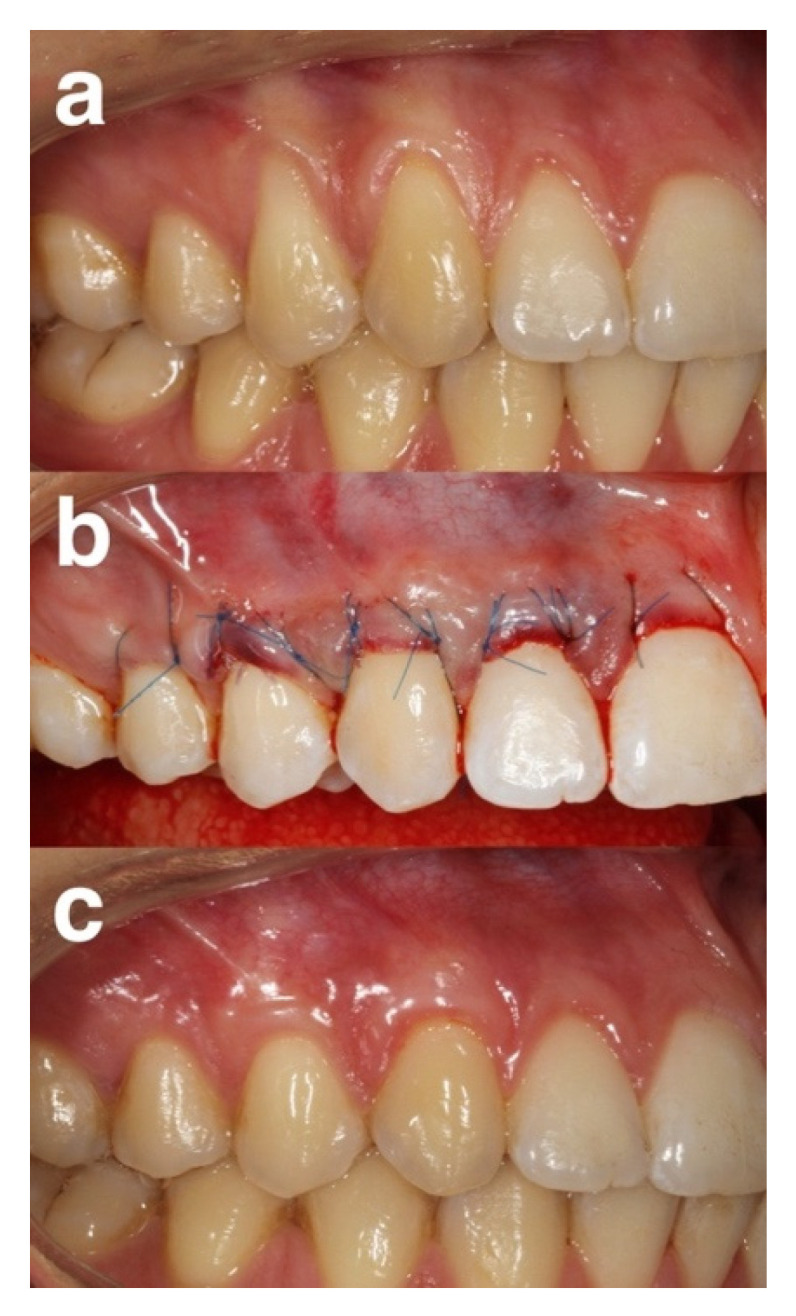
Representative case of the saline group (**a**) pre-operative view of gingival recessions. (**b**) immediate post-operative view. (**c**) 12 months post-operative view.

In the saline group, the root surfaces were burnished for 2 min using cotton pellets soaked in sterile saline and then rinsed with sterile saline (Figure 1). 

**Figure 2:**
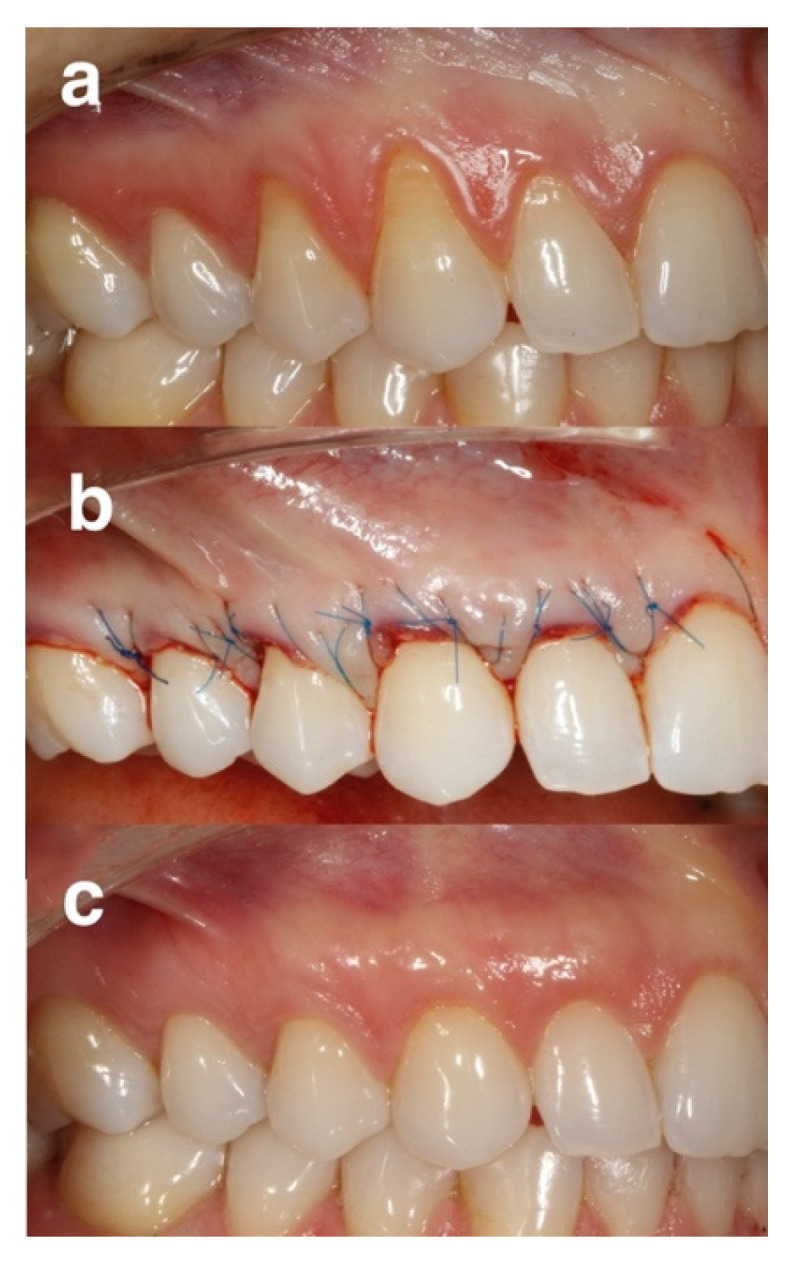
Representative case of the EDTA group: (**a**) pre-operative view of gingival recessions, (**b**) immediate post-operative view, (**c**) 12 months post-operative view.

In the EDTA group the exposed root surfaces were conditioned for 2 min with 24% EDTA (PrefGel^®^, Straumann, Basel, Switzerland) and rinsed with sterile saline (Figure 2). 

**Figure 3:**
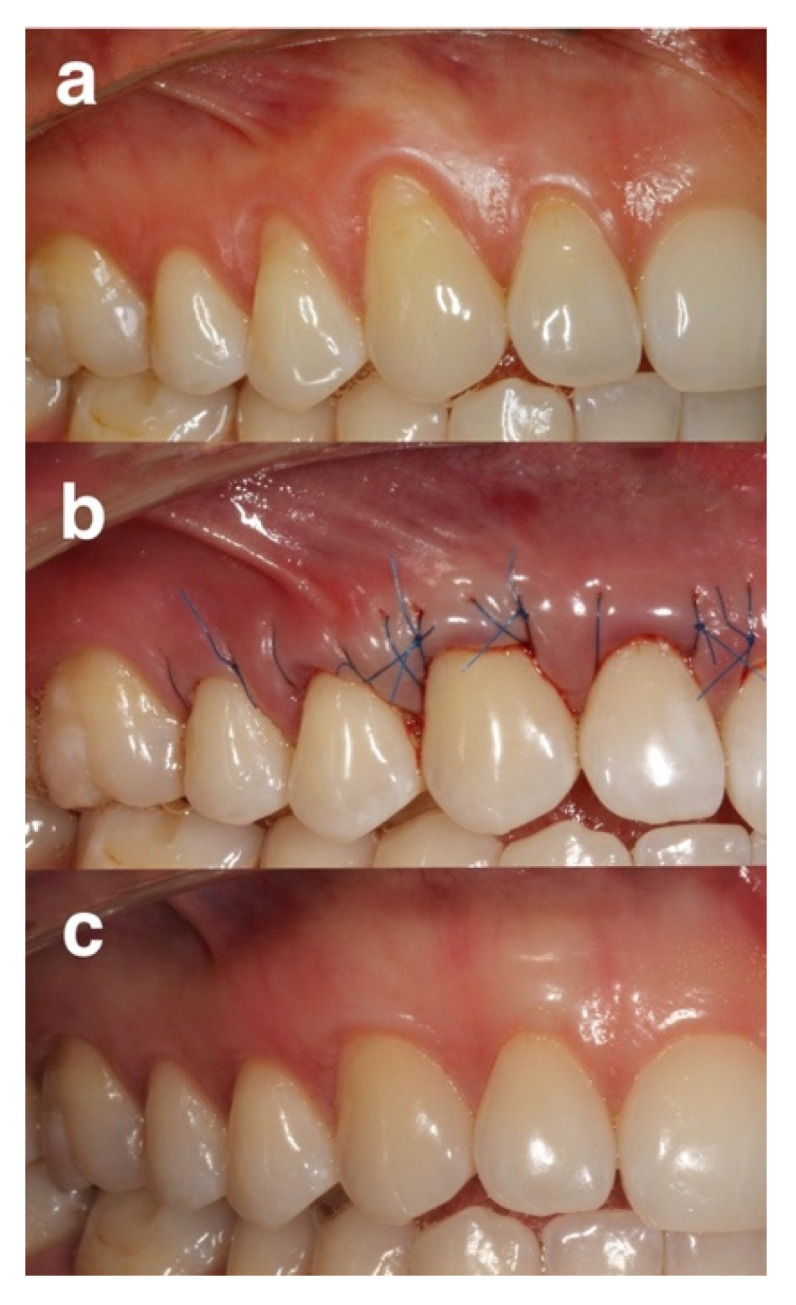
Representative group of the EDTA + EMD group: (**a**) pre-operative view of gingival recessions on control site, (**b**) immediate post-operative view, (**c**) 12 months post-operative view.

In the EMD group, root surfaces were conditioned for 2 min with 24% EDTA, rinsed with sterile saline, dried using cotton pellets and EMD (Emdogain^®^, Straumann, Basel, Switzerland) was applied (Figure 3). 

In the next step, SCTG was placed horizontally at or 1 mm below the CEJ and was stabilized with resorbable sling sutures (PGA Resorba 6/0 11 mm 3/8, RESORBA Medical GmBH, Nürnberg, Germany). Finally, the flap was coronally positioned and sutured with 6/0 non-resorbable monofilament sling sutures (Seralon 6/0 12 mm 3/8, Serag-Wiessner GmbH & Co, Naila, Germany). 

### 4.6. Post-Surgical Phase 

Patients were given 400 mg of ibuprofen and were asked to take the second dose 8 h later and an additional dose if required afterwards. They were told not to brush, floss, or chew in the treated area during the first 2 weeks, and to gently rinse the mouth using 0.2% chlorhexidine digluconate solution twice a day for 1 min during the first 14 days. Follow-up visits were scheduled at 1, 2 and 4 weeks and at 3, 6 and 12 months post-surgery. Each session involved reinforcement of oral hygiene instructions and professional plaque control. The sutures were removed 14 days after the surgery and patients were instructed in mechanical tooth cleaning of the surgical area with a soft post-surgical toothbrush using the roll technique. 

### 4.7. Statistical Analysis 

The analyses were carried out with 3.6.1 software (R Core Team 2021). The following calculations were performed: (1) recession reduction = GR0–GR12, (2) ARC = GR0–GR12/GR0 × 100%, (3) CAL gain = CAL0–CAL 12, (4) KTW gain = KTW12–KTW0, and (5) GT gain = GT12–GT0. Descriptive statistics were performed using mean values, standard deviations (SD), frequencies and percentages.

The data were analyzed in terms of compliance with normal distribution using the Shapiro–Wilk test. All study quantitative variables were normally distributed. Categorical variables were tested by Pearson’s chi-square test and continuous variables by the unifactorial analysis of variance (ANOVA). To analyze means of clinical parameters between baseline and 12 months after surgery, Student’s *t*-test was used. Unifactorial analysis of variance (ANOVA) was used to compare means between three treatment groups. Statistical significance was established for *p* < 0.05.

## Figures and Tables

**Table 1:** Characteristics for the study groups.

Variables	EDTA ^1^ + EMD ^2^ (*N* ^3^ = 20; *n* ^4^ = 70)	EDTA (*N* = 20; *n* = 72)	Saline (*N* = 20; *n* = 73)	*p*
Sex (*n*)				0.8170
Women	11	10	12	
Men	9	10	8	
Age (mean, SD ^5^)	28.47 (4.45)	29.02 (4.31)	28.56 (4.29)	0.6561
Tooth type (*n*)				0.9996
Incisors	15	16	16	
Canines	16	17	16	
Premolars	33	32	35	
Molars	6	7	6	
Tooth position (*n*)				0.9309
Maxillary teeth	54	57	56	
Mandibular teeth	16	15	17	
Type of GR ^6^ according to Cairo (*n*, %)				0.9384
RT ^7^1	47 (67.14)	49 (68.06)	51 (69.86)	
RT2	23 (32.86)	23 (31.94)	22 (30.14)	

^1^ EDTA ethylenediaminetetraacetic acid, ^2^ EMD enamel matrix derivative, ^3^ *N* number of patients, ^4^ *n* number of defects, ^5^ SD standard deviation, ^6^ GR gingival recession, ^7^ RT recession type.

**Table 2:** Clinical parameters (mean and standard deviation) at baseline and 12 months after surgery.

KERRYPNX	Baseline	12 Months	*p*
GR ^1^ EDTA ^2^ + EMD ^3^ (mm)	1.98 (1.11)	0.21 (0.45)	<0.0001 *
GR EDTA	1.82 (1.23)	0.26 (0.72)	<0.0001 *
GR Saline	1.78 (1.42)	0.22 (0.48)	<0.0001 *
*p*	0.4163	0.8761	
ARC ^4^ EDTA + EMD (%)		94.00 (20.12)	-
ARC EDTA		89.08 (31.76)	-
ARC Saline		91.1 (23.33)	-
*p*	-	0.8871	
CRC ^5^ EDTA + EMD (%)		64 (91.43)	-
CRC EDTA		65 (90.28)	-
CRC Saline		65 (89.11)	-
*p*	-	0.9743	
GR red ^6^ EDTA + EMD (mm)		1.78 (0.99)	-
GR red EDTA		1.56 (1.18)	-
GR red Saline		1.65 (1.25)	-
*p*	-	0.3029	
RW ^7^ EDTA + EMD (mm)	2.99 (1.33)	0.56 (1.23)	<0.0001 *
RW EDTA	2.76 (1.87)	0.52 (1.26)	<0.0001 *
RW Saline	2.45 (1.25)	0.54 (1.25)	<0.0001 *
*p*	0.3452	0.8981	
PPD ^8^ EDTA + EMD (mm)	1.47 (0.52)	1.76 (0.69)	0.0204 *
PPD EDTA	1.45 (0.59)	1.66 (0.68)	0.0342 *
PPD Saline	1.44 (0.50)	1.76 (0.71)	0.0198 *
*p*	0.8672	0.3481	
CAL ^9^ EDTA + EMD (mm)	2.56 (1.59)	1.22 (0.67)	0.0049 *
CAL EDTA	2.66 (1.65)	1.33 (0.78)	0.0104 *
CAL Saline	2.43 (1.62)	1.45 (0.88)	0.0183 *
*p*	0.5651	0.0387 *	
CAL gain EDTA + EMD (mm)		2.13 (1.12)	-
CAL gain EDTA		1.45 (1.10)	-
CAL gain Saline		1.32 (1.03)	-
*p*	-	0.0216 *	
KTW ^10^ EDTA + EMD (mm)	2.75 (1.33)	3.51 (1.31)	<0.0001 *
KTW EDTA	3.01 (1.32)	3.67 (1.02)	0.0018 *
KTW Saline	2.89 (1.37)	3.55 (1.22)	0.0119 *
*p*	0.2210	0.8101	
KTW gain EDTA + EMD (mm)		0.76 (0.99)	-
KTW gain EDTA		0.79 (1.01)	-
KTW gain Saline		0.79 (1.00)	-
*p*	-	0.4409	
GT ^11^ EDTA + EMD (mm)	1.16 (0.34)	2.05 (0.62)	<0.0001 *
GT EDTA	1.33 (0.47)	1.93 (0.63)	<0.0001 *
GT Saline	1.25 (0.33)	1.81 (0.52)	<0.0001 *
*p*	0.0545	0.3166	
GT gain EDTA + EMD (mm)		0.66 (0.55)	-
GT gain EDTA		0.63 (0.57)	-
GT gain Saline		0.54 (0.51)	-
*p*	-	0.6693	

^1^ GR gingival recession height, ^2^ EDTA ethylenediaminetetraacetic acid, ^3^ EMD enamel matrix derivative, ^4^ ARC average root coverage, ^5^ CRC complete root coverage, ^6^ GR red—gingival recession reduction, ^7^ RW gingival recession width, ^8^ PPD probing pocket depth, ^9^ CAL clinical attachment level, ^10^ KTW keratinized tissue width, ^11^ GT gingival thickness, * statistically significant (*p* ≤ 0.05).

**Table 3:** Evaluation of esthetic outcomes after 12 months—mean (standard deviation).

	GM ^3^	MTC ^4^	STT ^5^	MGJ ^6^	GC ^7^	RES ^8^
EDTA^1^ + EMD ^2^	5.62 (1.01)	0.98 (0.11)	0.95 (0.16)	0.98 (0.32)	1.00 (0.00)	9.65 (0.97)
EDTA	5.50 (1.07)	0.83 (0.21)	0.83 (0.21)	0.91 (0.33)	0.89 (0.30)	8.88 (1.22)
Saline	5.47 (1.11)	0.81 (0.20)	0.81 (0.23)	0.90 (0.35)	0.84 (0.31)	8.81 (1.30)
*p*	0.7982	0.0143 *	0.0264 *	0.12414	0.0187 *	0.0091 *

^1^ EDTA ethylenediaminetetraacetic acid, ^2^ EMD enamel matrix derivative, ^3^ GM gingival margin, ^4^ MTC marginal tissue contour, ^5^ STT soft tissue texture, ^6^ MGJ muco-gingival junction alignment, ^7^ GC gingival color, ^8^ RES Root Coverage Esthetic Score, * statistically significant (*p* ≤ 0.05).
